# CRISPR/Cas9 mediated knockout of *rb1* and *rbl1* leads to rapid and penetrant retinoblastoma development in *Xenopus tropicalis*

**DOI:** 10.1038/srep35264

**Published:** 2016-10-14

**Authors:** Thomas Naert, Robin Colpaert, Tom Van Nieuwenhuysen, Dionysia Dimitrakopoulou, Jannick Leoen, Jurgen Haustraete, Annekatrien Boel, Wouter Steyaert, Trees Lepez, Dieter Deforce, Andy Willaert, David Creytens, Kris Vleminckx

**Affiliations:** 1 Developmental Biology Unit, Department of Biomedical Molecular Biology, Ghent University, Ghent, Belgium; 2 Inflammation Research Center, VIB, Ghent, Belgium; 3 Center for Medical Genetics, Ghent University and Ghent University Hospital, Ghent, Belgium; 4 Laboratory for Pharmaceutical Biotechnology, Ghent University, B-9000 Ghent, Belgium; 5 Department of Pathology, Ghent University and Ghent University Hospital, Ghent, Belgium

## Abstract

Retinoblastoma is a pediatric eye tumor in which bi-allelic inactivation of the *Retinoblastoma 1* (*RB1*) gene is the initiating genetic lesion. Although recently curative rates of retinoblastoma have increased, there are at this time no molecular targeted therapies available. This is, in part, due to the lack of highly penetrant and rapid retinoblastoma animal models that facilitate rapid identification of targets that allow therapeutic intervention. Different mouse models are available, all based on genetic deactivation of both *Rb1* and *Retinoblastoma-like 1* (*Rbl1*), and each showing different kinetics of retinoblastoma development. Here, we show by CRISPR/Cas9 techniques that similar to the mouse, neither *rb1* nor *rbl1* single mosaic mutant *Xenopus tropicalis* develop tumors, whereas *rb1*/*rbl1* double mosaic mutant tadpoles rapidly develop retinoblastoma. Moreover, occasionally presence of pinealoblastoma (trilateral retinoblastoma) was detected. We thus present the first CRISPR/Cas9 mediated cancer model in *Xenopus tropicalis* and the first genuine genetic non-mammalian retinoblastoma model. The rapid kinetics of our model paves the way for use as a pre-clinical model. Additionally, this retinoblastoma model provides unique possibilities for fast elucidation of novel drug targets by triple multiplex CRISPR/Cas9 gRNA injections (*rb1* + *rbl1* + *modifier gene*) in order to address the clinically unmet need of targeted retinoblastoma therapy.

Retinoblastoma (RB) is the most common pediatric tumor of the developing retina^1^. The initiating mutations are lesions in the *Retinoblastoma 1* (*RB1*) tumor suppressor gene^2^. Statistical study of clinical retinoblastoma has led to the establishment of the two-hit hypothesis, stating that both *RB1* alleles must be mutated in order for retinoblastoma to develop^3^,^4^. Retinoblastoma can thus develop either in a heritable manner, due to a germline *RB1* mutation followed by a somatic *RB1* inactivation, or in a sporadic manner due to two independent somatic *RB1* mutations. *RB1* inactivation is also implicated in the initiation of other neoplasms including osteosarcoma, soft tissue cancer (spindle cell liposarcoma and atypical spindle cell lipoma) and small cell lung cancer, often in combination with *TP53* mutations^5^,^6^,^7^,^8^. Additionally, patients with germ-line *RB1* mutations are at risk of developing trilateral retinoblastoma, a pediatric intracranial neuroblastic tumor^9^,^10^.

While treatment of unilateral retinoblastoma by modern therapies is usually curative, treatment of bilateral retinoblastoma with the aim to obtain survival, eye salvage and preservation of vision still represents a major challenge^11^. Treatment regimens have, however, recently improved and now routinely incorporate novel strategies such as ophthalmic artery chemosurgery, intravitreous chemotherapy and aggressive focal therapies^12^. However, no targeted molecular therapies exist within the clinic for treatment of retinoblastoma and this slow progress can be partly attributed to the lack of preclinical models that allow for rapid identification of druggable targets involved in the etiology of retinoblastoma^13^.

Unlike the human situation, mice heterozygous for *Rb1* do not develop retinoblastoma. Instead they develop a multiple endocrine neoplasia syndrome manifested by development of pituitary and thyroid tumors^14^. Homozygous *Rb1* mutants manifest embryonic lethality due to abnormalities in neural and hematopoietic development^15^,^16^. Murine retinoblastoma was first observed in chimeric animals lacking both *Rb1* and *Retinoblastoma-like 1* (*Rb1*/*Rbl1*), however the low recovery of viable chimeras precluded the generation of study-sized populations of retinoblastoma-bearing mice^17^. Current breedable murine retinoblastoma models depend on conditional deletion of the *Rb1* gene using cre-transgenics on a *Rbl1*^−/−^ (with or without a *p53*^−/−^ or *PTEN*^−/−^) genetic background^18^,^19^,^20^,^21^. Whilst these latter cre-transgenic driven models exhibit high penetrance of retinoblastoma development, the latency is still relatively long (e.g. 100 ± 42.3 days in the *Chx10-Cre*; *Rb1*^*Lox/Lox*^; *Rbl1*^−/−^; *p53*^−/−^ model), which has an impact on pre-clinical drug screening efforts^13^,^22^.

We have recently shown that targeted genome editing techniques, such as TALENs and CRISPR/Cas9, allow for cheap high-throughput gene knock-out approaches and modeling of cancer syndromes in the aquatic amphibian model *Xenopus tropicalis*^23^. Importantly, unlike *X. laevis* and zebrafish, *X. tropicalis* has a true diploid genome that may facilitate modeling human genetic diseases, including cancer^24^,^25^.

Here we describe the first genuine genetic CRISPR/Cas9 mediated cancer model in *X. tropicalis*. We show rapid and penetrant retinoblastoma development in *X. tropicalis*, by co-targeting of *rb1* and *rbl1* with multiplex CRISPR/Cas9, closely recapitulating the histopathological hallmarks of clinical retinoblastoma. This model provides an interesting platform for pre-clinical drug screening efforts. Additionally this rapid model can be exploited for fast exploration of the impact of inactivating modifier or effector genes on the resulting phenotype by CRISPR/Cas9 multiplexing.

## Results

### *Rb1* mosaic mutants develop normally and lack tumor formation

Two-cell stage *X. tropicalis* embryos were injected unilaterally with *rb1* coding region 1 gRNA (*rb1*^*cr1*^) and recombinant Cas9 protein. Embryos were grown until stage 46 and pools of embryos were lysed and genomic DNA was extracted. The targeted region was amplified and successful insertion-deletion (INDEL) mutations in the *rb1* locus (4%) were confirmed by targeted deep sequencing. Please note that due to the unilateral injection setup, this implies that one side across the ventral midline of the tadpole or froglet is 8% mosaic mutant, whereas the other side is essentially wild-type. Efficiencies and variant calls for all next-generation sequencing experiments can be found as Supplementary Table S1.

We did not observe any histological or proliferation abnormalities (by proliferating cell nuclear antigen/PCNA staining) in the eyes of 7 days old mosaic *rb1* mutant tadpoles (not shown). Furthermore, the eyes of four months old adult *rb1* mosaics showed no abnormalities in retinal structure. *Rb1* mosaics were raised up to sixteen months of age and none (n = 13) developed retinoblastoma distinguishable by gross examination. Together, these data indicate that, in contrast to the human situation, but in line with studies in the mouse, bi-allelic inactivation of the *rb1* gene is insufficient for retinoblastoma development in *Xenopus*. Although the efficiencies of gene disruption were relatively low, we believe that if *rb1* bi-allelic mutation was sufficient to initiate tumorigenesis, tumors would have been detected. This due to the expected selective growth advantage of this hypothetical population of *rb1* mutant tumor cells and the large original cohort size (n = 50).

### *Rbl1* mosaic mutants develop normally and lack tumor formation

Motivated by the studies in mice where it was shown that bi-allelic mutations in both the *Rb1* and *Rbl1* genes induced retinoblastoma, we wanted to investigate whether this was also the case in *Xenopus*. In order to ensure that *rbl1* mutant animals are tumor-free, two-cell stage *X. tropicalis* embryos were unilaterally injected with *rbl1* coding region 1 (*rbl1*^*cr1*^) gRNA. Embryos were grown until stage 46 and pools of embryos were lysed and genomic DNA was extracted. The targeted region was amplified and successful INDEL mutations in the *rbl1* locus (26%) were confirmed by targeted deep sequencing. No retinoblastoma or histopathological abnormalities were detected in the eyes of post-metamorphic froglets (aged 58 days; n = 3). *Rbl1* mosaic mutants (n = 5) were raised until 73 days of age, and none developed retinoblastoma distinguishable by gross examination.

### Retinoblastoma and brain tumors rapidly develop in mosaic *rb1/rbl1* mutant tadpoles and froglets

As we did not detect any retinoblastoma after inactivation of either *rb1* or *rbl1*, we next performed multiplex genome editing by co-injections of two independent pairs of *rb1* and *rbl1* gRNAs. By employing two independent pairs of gRNAs, we circumvent possible development of phenotypes due to off-target effects of the CRISPR/Cas9 system, because it is highly unlikely that two distinct gRNAs will have an overlap in off-target profiles. Two-cell stage *X. tropicalis* embryos were unilaterally injected with either *rb1*^*cr1*^ and *rbl1*^*cr1*^ gRNA or *rb1*^*cr2*^ and *rbl1*^*cr2*^ gRNA together with recombinant Cas9 protein. Embryos were grown until stage 46 and pools of embryos were lysed and genomic DNA was extracted. Successful targeting of the loci was confirmed by targeted deep sequencing in the first setup (26% *rb1* and 28% *rbl1*) as well as the second setup (9% *rb1* and 10% *rbl1*).

**Figure 1:**
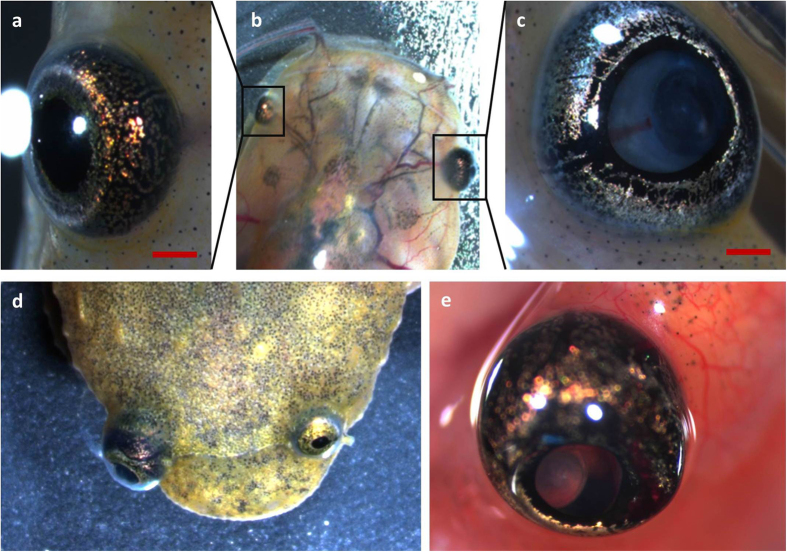
*Retinoblastoma 1* and *retinoblastoma-like 1* CRISPR/Cas9 injected *Xenopus tropicalis* develop retinoblastoma. **(a–c)** Development of unilateral retinoblastoma can be externally observed in tadpoles injected with *rb1* and *rbl1* gRNAs, when comparing the eye on the uninjected side with the eye on the injected side in this tadpole. Leukocoria and ectopia lentis are apparent. **(d)** Unilateral retinoblastoma in a froglet. **(e)** Externally visible tumor-associated neovasculature.

Mosaic double *rb1*/*rbl1* knockout tadpoles (MDKO tadpoles) rapidly developed externally visible eye tumors as early as 36 days post-injection. In total, MDKO tadpoles and frogs developed retinoblastomas ranging from smaller neoplasms, distinguishable only by increased size of one eye respectively to the other, to large retinoblastoma exhibiting leukocoria, ectopia lentis and tumor-associated neovasculature (Figs 1 and S1). Retinoblastomas exhibited rapid tumor progression, approximately doubling in size in seven days after being detected by gross examination. Some tadpoles also exhibited neurological symptoms such as abnormal swimming behavior, lethargy and reduced response to stimuli such as sound or touch. The animals were euthanized as soon as they showed clear signs of discomfort. The complete heads of tadpoles were dissected, decalcified and processed for histological analysis. Unilateral retinoblastoma was detected in 53% (n = 13; median 120 days) and 73% (n = 15; median 69 days) of the MDKO tadpoles for the *rb1*^*cr1*^/*rbl1*^*cr1*^ and *rb1*^*cr2*^/*rbl1*^*cr2*^ gRNA injections, respectively.

These results are counterintuitive given the lower INDEL efficiency for the *rb1*^*cr2*^/*rbl1*^*cr2*^ set up. However, we postulate that these differences in retinoblastoma incidence are the consequence of the distinct targeting sites of the *rbl1*^*cr1*^ and *rbl1*^*cr2*^ gRNAs. The *rbl1*^*cr2*^ gRNA targets the pocket domain of the Rbl1 protein, a region of functional importance by binding E2F transcription factors, whilst the *rbl1*^*cr1*^ gRNA does not target a functional domain. We believe that, in this region, small in-frame deletions or insertions, could still perturb protein function.

In addition to the retinal tumors, we were able to demonstrate brain neoplasms in 11% (n = 13) and 13% (n = 15) for *rb1*^*cr1*^/*rbl1*^*cr1*^ and *rb1*^*cr2*^/*rbl1*^*cr2*^ setups, respectively. These brain tumors can be the consequence of either locally invasive retinoblastoma or may represent discrete entities as intracranial midline tumors associated with the pineal gland (pinealoblastoma, trilateral retinoblastoma). Unfortunately, we were unable to confidently discriminate between these two possibilities, considering we have no tool to track the origin of these neoplasms.

Moreover, we have detected several other types of neoplasia in MDKO tadpoles, in particular small cell lung cancer, a choroid plexus neoplasm and one case of a hibernoma (Fig. S2). None of these were however detected more than once, and we thus consider MDKO tadpoles to be predisposed to several cancer types dependent on additional somatically acquired mutations.

### Histological analysis confirms the presence of retinoblastoma histopathology

**Figure 2:**
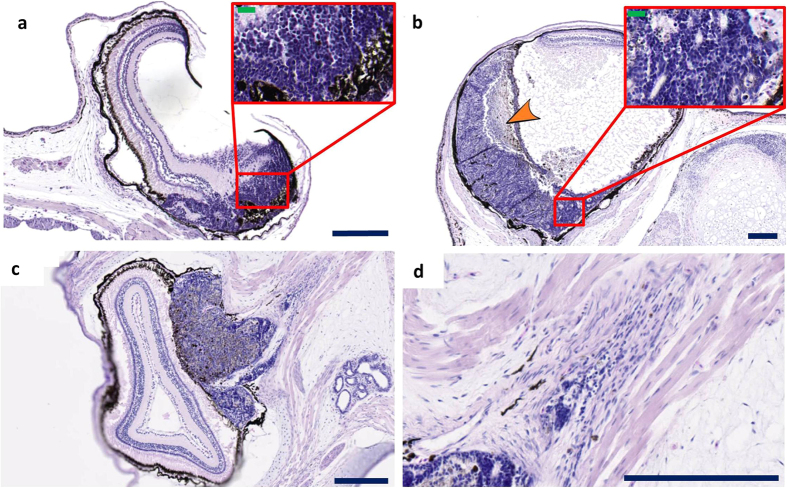
Histopathology of retinoblastomas. **(a–d)** Examples of retinoblastomas observed in tadpoles and froglets injected with *rb1* and *rbl1* gRNAs. Under low magnification, retinoblastoma appears as a large basophilic mass with pink and purple foci. These tumor masses arise from and destroy the retina and can either fill part of the vitreous cavity, thus following an endophytic growth pattern **(a,b)** including necrotic areas (orange arrowhead), or can grow in the outer layers of the retina (exophytic growth pattern) **(c)**. The poorly differentiated neuroblastic cells appear blue because they have intensely basophilic nuclei and scanty cytoplasm (‘small blue round cell tumor’) (inset **a,b**). The poorly differentiated neuroblastic cells show varying degrees of retinal differentiation with formation of (Homer Wright) rosettes. The tumors with exophytic growth can invade in the optic nerve (**d**). Scale bar sizes as follows; Blue scale bars are 200 μm. Green scale bars are 20 μm. Sections have been cut longitudinal.

**Figure 3:**
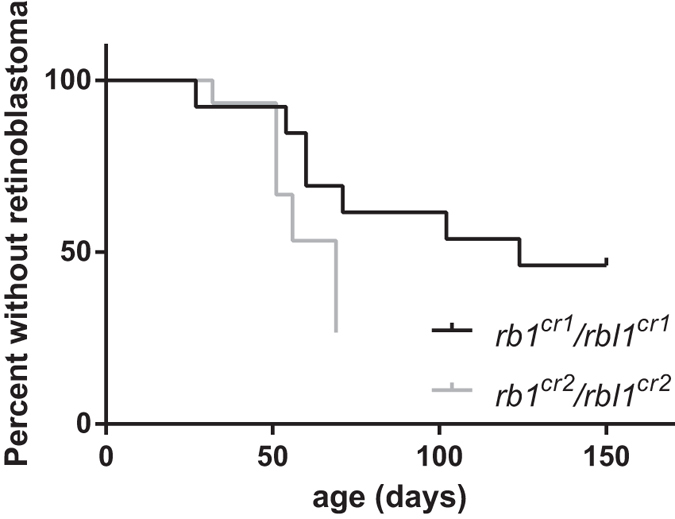
Appearance of retinoblastoma. Graph indicating occurrence (and time-point) of retinoblastoma detection. Detection points either correspond to euthanasia of moribund tadpole/froglet with apparent retinoblastoma or with an end-point of the experiment (127 days for *rb1*^cr1^/*rbl1*^cr1^ and 69 days for *rb1*^cr2^/*rbl1*^cr2^). Each data point was validated by histopathological validation of the malignancy.

**Figure 4:**
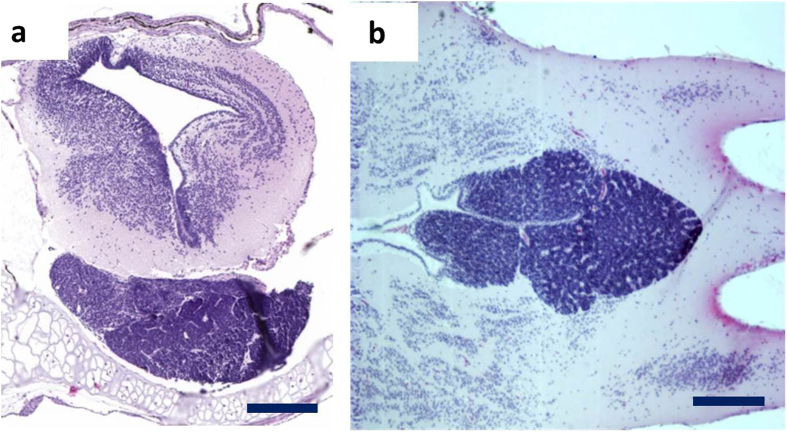
Histopathology of brain tumors. (**a,b**) Low magnification shows large basophilic poorly differentiated small blue round cell tumors, which are located across the midbrain and cranial midline. Due to the location we believe we show a pinealoblastoma (trilateral retinoblastoma) in (**b)**. Scale bar sizes as follows; Scale bars are 200 μm. Section (**a**) has been cut longitudinal and section (**b)** has been cut transversal.

Histology revealed retinoblastoma appearing as a large basophilic mass that arises from, and destroys, the retina (Fig. 2a,b). It was observed that the retinoblastoma can seed into the vitreous cavity (endophytic growth pattern) and can exhibit areas of necrosis (orange arrowhead in Fig. 2b). Moreover, we also detected retinoblastoma arising from the outer layers of the retina exhibiting an exophytic growth pattern (Fig. 2c). Higher magnification investigation revealed poorly differentiated neuroblastic cells that appear blue due to intensely basophilic nuclei and scant cytoplasm (small-blue-round-cell tumor) (Fig. 2a,b insets). Optic nerve invasion of small-blue-round-cells was also observed, demonstrating local spread of tumor cells (Fig. 2c,d). Ultimately, we plotted occurrence (and time-point) of retinoblastoma development by histopathological analysis (Fig. 3). Histological analysis of MDKO tadpoles, or froglets, also revealed large basophilic poorly differentiated small-blue-round-cell brain tumors, which were located across the midbrain and cranial midline (Fig. 4). As mentioned before, our model does not allow distinguishing between primary brain tumors and retinoblastoma metastasis. Nevertheless, due to the location of a subset of brain neoplasms, which were located right across the cranial midline, we believe that we detect pinealoblastoma or trilateral retinoblastoma (Fig. 4b). Additionally, we also detected a pinealoblastoma in an animal which did not show retinoblastoma development upon histopathological assessment of the eyes.

### The neoplasms show extensive hyper proliferation

**Figure 5:**
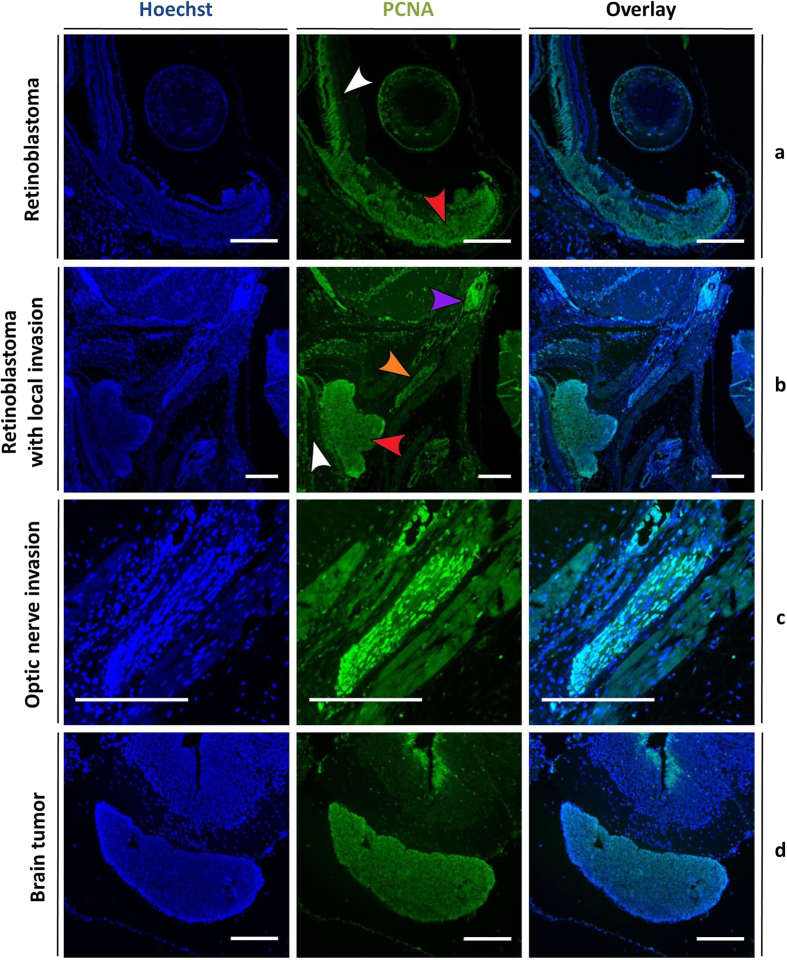
Staining for a proliferation marker demonstrates aggressive growth characteristics of tumor cells in *rb1*^cr1^/*rbl1*^cr1^ MDKO tadpoles. Hoechst (left panels) and proliferating cell nuclear antigen (PCNA) (middle panels) double staining, with overlay in right panel. (**a,b**) PCNA staining is detected within the retinoblastoma (red arrowhead) whilst the adjacent normal retina remains quiescent (white arrowhead). In (**b**), tumor cells invading into the optic nerve (orange arrowhead) and a more distant metastasis (purple arrow) can be distinguished. (**c**) Higher magnification of the optic nerve clearly reveals PCNA positive tumor cells. (**d**) PCNA staining is uniformly detected within the brain tumor, whereas the surrounding normal brain tissue remains quiescent, with the exception of the subventricular zone. Scale bars are 200 μM.

To further examine proliferation characteristics of the retinoblastoma and brain neoplasms of *rb1*^*cr1*^/*rbl1*^*cr1*^ MDKO tadpoles, we performed PCNA immunohistochemistry. Nuclear PCNA staining was uniformly detected in the retinoblastoma nuclei whilst the adjacent retina remained quiescent (Fig. 5a,b). Additionally, PCNA positive tumor cells could be detected in a brain tumor and within the optic nerve of the injected side (Fig. 5), but not in the optic nerve of a wild-type eye (Fig. S3). Higher magnification of the invaded optic nerve clearly shows cycling cells within the optic nerve (Fig. 5). Next to this, PCNA staining was also uniformly detected in the brain tumor whereas the surrounding brain remained quiescent, with the exception of actively cycling cells in the subventricular zone (Fig. 5). PCNA immunohistochemistry on *rb1*^*cr2*^/*rbl1*^*cr2*^ MDKO tadpoles revealed identical proliferation characteristics of the neoplasms (Fig. S4). These immunohistochemical data validate the aggressive growth characteristics of the detected retinoblastoma, exhibiting local invasion and possibly distant metastasis.

### Neoplasms are characterized by double bi-allelic *rb1* and *rbl1* mutations

In order to confirm that the retinoblastoma and brain tumors are clonal outgrowths of cells carrying mutations in both the *rb1* and *rbl1* gene, we dissected an eye exhibiting retinoblastoma development and a normal looking eye from the injected side of a MDKO tadpole (Fig. S5). By targeted deep sequencing we showed that the normal appearing eye had *rb1* (26%) and *rbl1* (22%) INDELs at levels similar to the general genome editing efficiency in the setup. In contrast, the retinoblastoma showed enrichment of INDELs in both *rb1* (80%) and *rbl1* (74%) loci. To further validate this, laser capture microdissection (LCM) was used to isolate retinoblastoma cells, adjacent normal retina, retina from the non-injected side and brain tumor (Fig. S6) from a histological section. Genomic DNA was extracted from these isolates, followed by targeted deep sequencing of the *rb1*^*cr1*^and *rbl1*^*cr1*^ loci. The retinoblastoma was strongly enriched in two *rb1* frameshift deletions (85%; Δ52 and Δ7) and an *rbl1* insertion (58%; +36). Note that these percentages do not reach 100% due to presence of pre-existing cells, blood cells and immune cells within the microdissected tissue. In contrast, the adjacent normal retina showed a lower level of *rb1* deletion (16%; Δ30 and Δ19) and *rbl1* insertion (50%; +1) . Additionally, the non-injected eye shows not a single INDEL in the *rb1* locus and only a very basal level of *rbl1* deletion (5%; Δ15). The brain neoplasm, however, was also heavily enriched for *rb1* frameshift deletion (89%; Δ52 and Δ7) and *rbl1* insertion (97%; +32). In order to validate this data, retinoblastoma and brain tumor were microdissected from a second tadpole from this cohort, attempting not to transfer any pre-existent cells (i.e. retinal pigment epithelium) (Fig. S7). By this method, almost universal *rb1* and *rbl1* INDELs (88% to 100%) could be demonstrated within the neoplasms (Table S1). This data implies that the neoplasms developing within the *rb1/rbl1* MDKO tadpoles are discrete outgrowths of clones characterized by double bi-allelic mutations in *rb1* and *rbl1* as a consequence of the CRISPR/Cas9 genome editing. We observed, in some cases, only a single insertion in the *rbl1* gene and this may indicate that loss of heterozygosity (LOH) occurred in the tumor. This needs further evaluation, but interestingly the occurrence of a very specific LOH in both the retinoblastoma as well as the brain tumor might indicate that this brain tumor is in fact clinically invaded retinoblastoma.

### Triple multiplex gene disruption, facilitating therapeutic target identification, is possible within *rb1* ^*cr2*^ */rbl1* ^*cr2*^ *Xenopus* retinoblastoma model

In order to perform therapeutic target identification in our model, we need to provide proof-of-principle for triple multiplex genome editing in *Xenopus tropicalis*. For this, we chose to target the spleen associated tyrosine kinase *(SYK)* gene, which was recently identified as a promising potential therapeutic target for molecularly targeted treatment of retinoblastoma^26^. Specifically, it was shown that the *SYK* gene is epigenetically deregulated and expressed at high levels in retinoblastoma thus preventing programmed cell death through MCL1^26^. However, *Syk* was not found to be epigenetically deregulated in murine retinoblastomas and subsequently the impact of a direct genetic knockout of *SYK* on retinoblastoma tumorigenesis has not been investigated yet^27^. We unilaterally injected two cell stage *Xenopus tropicalis* embryos with *rb1*^*cr2*^*, rbl1*^*cr2*^, *syk* gRNA and recombinant Cas9 protein. Embryos were grown until stage 46 and pools of embryos were lysed and genomic DNA was extracted. We were able to demonstrate, by targeted deep sequencing, efficient genome modification of the *rb1* (13%)*, rbl1* (10%) and *syk* (8%) loci. Please note that these efficiencies are more or less similar for all three loci. As this experiment only served as a pilot proof-of-principle, it only encompassed a very small number of animals (n = 10) and further work is evidently needed and will be performed to accurately identify the impact of *syk* knockout on retinoblastoma tumorigenesis.

## Discussion

We describe a novel retinoblastoma model in *Xenopus tropicalis* that accurately recapitulates the clinical symptoms and the histopathological hallmarks of retinoblastoma. Progression also seems to closely mimic the clinical situation. This as we have detected in MDKO animals, small tumors, still confined to the retina, up to tumors exhibiting extensive vitreous chamber seeding and local invasion within the optic nerve. We provide some evidence that this invasion can culminate in intracranial metastasis. Additionally, we suspect, due to location, the presence of trilateral retinoblastoma in at least one of the analyzed MDKO tadpoles and froglets. This low level of occurrence correlates well with the low clinical incidence of trilateral retinoblastoma^28^,^29^.

Setting up large clinical trials to validate novel treatment strategies is very hard due to the fact that the incidence of retinoblastoma is low and the curative rates obtained by established therapies are high, at least for unilateral retinoblastoma, which implies that patient recruitment for clinical trials is very challenging^19^. This, in turn, makes setting up large clinical trials to validate novel treatment strategies arduous. Rapid and highly penetrant pre-clinical disease models for retinoblastoma are therefore extremely valuable for drug validation efforts, which can then be translated to front-line treatments^30^.

Animal models for retinoblastoma have been described before in both mice and zebrafish. An orthotopic retinoblastoma transplantation model in zebrafish exhibiting invasiveness and metastasis has been used for screening anti-cancer compounds^31^,^32^. Besides, it was shown that six established retinoblastoma mouse strains, all based on the simultaneous inactivation of the *Rb1* and *Rbl1* genes, exhibited extensive overlap and intermixing of their gene expression profile^22^. The authors subsequently demonstrated through principal component analysis that most of the difference between the gene profiles of human and mouse retinoblastoma cells were within a single dimension. This implies that the *Chx10-Cre; Rb1*^*Lox/Lox*^; *Rbl1*^−/−^ mice model, and the five others, are suitable models closely mimicking the molecular background of clinical retinoblastoma. By extension, this strongly suggests that modeling retinoblastoma by knocking out *Rb1* and *Rbl1* in mice is a suitable approach, which we expect to be similar in other animals models, such as *Xenopus tropicalis*.

We believe that our retinoblastoma model, the first genuine genetic retinoblastoma model in a non-mammal and the first CRISPR/Cas9 mediated cancer model in *Xenopus tropicalis*, can provide several unique advantages. Firstly, as a genuine genetic model it competes well with the orthotopic zebrafish models. Secondly, our model generated with the second gRNA pair (*rb1*^*cr2*^ and *rbl1*^*cr2*^), with median time of 69 days until retinoblastoma development, shows faster tumor emergence kinetics than the established *Chx10-Cre; Rb1*^*Lox/Lox*^*; Rbl1*^−/−^; *p53*^−/−^ model (Median time until RB development: 100 ± 42.3 days)^13^. Please note that the *Xenopus* median time until retinoblastoma development is counted as days post-fertilization, whilst with any mice model 21 days (gestation time) should be added to the reported median time. Considering that this mouse model has already been successfully employed for pre-clinical studies, this implies that our *Xenopus* model could also be used for such purposes^33^. Additionally, the extensive brood size and external development of *X. tropicalis*, coupled with the ease by which CRISPR/Cas9 can be introduced by standard micro-injection, allows generation of several hundreds of *rb1*/*rbl1* mosaic mutant animals in one mating. These cohort sizes easily surpass those feasible in murine studies, without the need for time-consuming breeding. Next to this, unilateral injection in two-cell *X. tropicalis* embryos provides the unique possibility to generate *Xenopus rb1*/*rbl1* mosaic mutant animals that exhibit essentially one wild-type eye and one mosaic mutant eye. This implies that in a drug screening effort, one can immediately assess the effect of the drug not only on the retinoblastoma, but also for toxicity on normal retinal cell homeostasis.

Finally and importantly, the presented *X. tropicalis* retinoblastoma model provides unique possibilities for fast exploration of the impact of inactivating modifier (or effector) genes on cancer development by multiplex CRISPR/Cas9 gRNA injections. This in contrast to low latency mice models (*e.g. Chx10-Cre; Rb1*^*Lox/Lox*^; *Rbl1*^−/−^; p53^−/−^) where targeting an additional locus, on top of the existing modified ones, becomes a difficult feat to accomplish from a breeding and locus segregation perspective. Note that *X. tropicalis* is a true diploid and there is very high synteny between the human and the *X. tropicalis* genomes, which greatly facilitates the identification of the frog orthologs for most human genes of interest^24^. We have previously shown that multiplexed gene targeting in developing *Xenopus* tumors is possible^23^. Moreover, we have shown here that we are able to target three distinct loci (*rb1, rbl1* and *syk*) by triple multiplex CRISPR/Cas9 at genome editing levels that are more or less similar for each locus. Taken together, we thus hypothesize that retinoblastoma developing in these triple mutants should show INDELs for all three targeted loci, unless there is negative selection for modification of the modifier gene (Fig. S8). For this *rb1, rbl1* and a modifier are targeted by triple multiplex CRISPR/Cas9 and retinoblastomas should be (micro)dissected. If bi-allelic modifier gene mutations can be detected in (micro)dissected retinoblastoma, this indicates that the modifier is not critical for tumor formation. The other possibility is that bi-allelic modifier gene mutations are never detected in (micro)dissected retinoblastoma. This provides strong evidence that the modifier is critical in retinoblastoma tumorigenesis, due to apparent negative selection. This implies, like stated above, that this modifier is critical in retinoblastoma tumorigenesis and that it might represent a very attractive novel drug target. The fact that such an experiment can already be performed in 2–3 months opens up opportunities for high-throughput therapeutic target identification. Such targeted drug discovery efforts can lead to fast and rational drug design and development of novel strategies for treatment of retinoblastoma.

We firmly believe that non-mammalian vertebrate cancer models like the one presented here, which profoundly recapitulate the clinical situation, will be key in order to have the biomedical community conform to the requests from the public and policymakers to reduce the amount of mammals used in pre-clinical studies. Whilst models with closer evolutionary distance to humans will always be necessary for validating studies performed in those models with further evolutionary distance, we feel that non-mammalian vertebrate models can provide significant advantages during earlier stages of drug discovery and for rapid therapeutic target identification.

## Material and Methods

### gRNA design and generation

*Rb1*^*cr1*^ gRNA was designed with the Doench, Hartenian algorithm^34^. *Rbl1*^*cr1*^ gRNA was designed with the CCTop tool^35^. *Rb1*^*cr2*^, *rbl1*^*cr2*^ and *syk* gRNAs were designed with the CRISPRScan algorithm^36^.

In this study, the following sequences were targeted. *Rb1*^*cr1*^ 5′-AGACAAACAAGGGAACGGGA-3′, *rb1*^*cr2*^ 5′-GCTGTATGATTGTGCTGTACCGG-3′, *rbl1*^*cr1*^ 5′-ATATTTCAAAACCCTCACG-3′, *rbl1*^*cr2*^5′-TGGGCTTGCGCGCTGATGTGGGG-3, *syk* 5′-TGGGTAGGAGGTGCTGGACATGG-3′. A PCR-based strategy for generating DNA templates for *in-vitro* gRNA transcription was employed as described before^37^. The 5′ primer contains the target sequence and has the form 5′- GAAATTAATACGACTCACTATAGG(N)_16–20_ GTTTTAGAGCTAGAAATAGC-3′ whilst the 3′ primer is common to all gRNA templates and is as follows 5′AAAAGCACCGACTCGGTGCCACTTTTTCAAGTTGATAACGGACTAGCCTTATTTTAACTTGCTATTTCTAGCTCTAAAAC-3′. Assembly reaction was performed with Phusion high-fidelity polymerase (Thermo-scientific). DNA templates were purified by phenol-chloroform extraction/NaOAc precipitation. *In-vitro* transcription of the gRNA was performed with either the MEGAshortscript™ T7 Transcription Kit (ThermoFisher Scientific) or HiScribe™ T7 High Yield RNA Synthesis Kit (New England Biolabs) and gRNAs were purified by phenol-chloroform extraction/NH_4_OAc precipitation. Concentrations were determined by Nanodrop (Thermo-Scientific).

### Recombinant NLS-Cas9-NLS generation

pX330-U6-Chimeric_BB-CBh-hSpCas9 was a gift from Feng Zhang (Addgene plasmid #42230)^38^. Cloning from this plasmid led to generation of pLHM36NLS(S)-Cas9(SP)-NLS(N). Subsequently, recombinant NLS-Cas9-NLS was expressed in the *Escherichia coli* strain BL21codon + pICA2 after transformation with pLHM36NLS(S)-Cas9(SP)-NLS(N) and purified. The pLHM36NLS(S)-Cas9(SP)-NLS(N) plasmid description, protein expression induction and protein purification strategies are shown in Supplementary data.

### *Xenopus tropicalis* microinjection

Wild-type *Xenopus tropicalis* female and male were primed with 20U and 10U PREGNYL© human chorionic gonadotropine (hCG) (Merck), respectively. Natural matings were set-up 2 days later, after boosting the female and male with 150U and 100U of hCG, respectively. Embryos were collected as described before^39^. Embryos were unilaterally injected at the two cell stage with 1nl of injection mix containing combinations of gRNA and NLS-Cas9-NLS (VIB Protein Service Facility, UGent) as shown in Supplementary Table S2. All experiments were approved by the Ethical Committee for Animal Experimentation from Ghent University, Faculty of Science and VIB-Site Ghent (Ethical Approval #2013-076). All methods were carried out in accordance with the relevant guidelines set out by this committee.

### DNA extraction and targeted deep sequencing

For analyzing the genome editing efficiency tadpoles, tissue or tumors were incubated overnight at 55 °C in lysis buffer (50 mM Tris pH 8.8, 1 mM EDTA, 0.5% Tween-20, 200 μg/ml proteinase K). For assessing total genome editing efficiency for a specific injection setup we pooled a minimum of 5 stage 46 tadpoles and performed lysis by proteinase K treatment. The locus of interest was amplified by PCR with the respective primer pairs as shown in Table S3. Targeted deep sequencing of PCR products was performed using a previously described workflow^40^,^41^. Indel frequency data and sequence variants for all targeted deep sequencing are shown in Supplementary Table S1.

### Imaging, histology and immunohistochemistry

All pictures of euthanized and living tadpoles were taken with a Carl Zeiss StereoLUMAR.V12 stereomicroscope. Complete heads of euthanized tadpoles were dissected, fixed in 4% PFA (paraformaldehyde) and decalcified by Morse’s solution (10% sodium citrate and 22.5% formic acid) for 7 hours up to overnight (depending on age of specimen). Tissue samples were subsequently dehydrated and embedded in paraffin. Tissue sections of 5 μM were cut by microtome, rehydrated and hematoxylin and eosin stained with a Varistain™ 24-4 Automatic Slide Stainer (Thermo-Scientific). Images were captured with an Axio Scan.Z1 (Zeiss, Germany). Images were acquired with a 20X Plan-Apochromat 0.8 NA dry objective, using a Hitachi HV-F202SCL camera. For PCNA immunohistochemistry, antigen retrieval was performed using the PickCell 2100-Retriever (ProteoGenix) in citrate buffer (10 mM citric acid, 0.1% Tween-20, pH 6). Slides were subsequently blocked with blocking buffer (3% goat serum, 1% BSA, 0.1% Tween-20) and incubated overnight with PCNA antibody (Clone PC10-Dako) diluted 1/500 in blocking buffer at 4 °C. Detection was performed with secondary goat anti-mouse dylight-594 and counter-staining was performed with Hoechst-33342. Sections were subsequently imaged using a Leica TCS LSI zoom confocal microscope.

### Genotyping of tumors by laser capture microdissection

Five μM sections containing tissues of interest were rehydrated, stained with hematoxylin and eosin and dehydrated to 100% EtOH. Cells/Tissues were microdissected by laser pressure catapulting (LPC) using a P.A.L.M. MicroBeam laser microdissection system (P.A.L.M. Zeiss Microlaser Technologies, Munich, Germany). DNA was purified from micro dissected cells with the QIAamp DNA Micro Kit (Qiagen), the appropriate genomic regions containing the CRISPR/Cas9 cut sites were PCR amplified and targeted deep sequencing was performed as described above.

## Additional Information

**How to cite this article**: Naert, T. *et al*. CRISPR/Cas9 mediated knockout of *rb1* and *rbl1* leads to rapid and penetrant retinoblastoma development in *Xenopus tropicalis*. *Sci. Rep*. **6**, 35264; doi: 10.1038/srep35264 (2016).

## Supplementary Material

Supplementary Information

Supplementary Data
