# Supplementary material for: CRISPR/Cas9 mediated knockout of rb1 and rbl1 leads to rapid and penetrant retinoblastoma development in Xenopus tropicalis
Source: Sci Rep. 2016 Oct 14;6:35264. doi: 10.1038/srep35264 (PMC5064383; doi:10.1038/srep35264)
Supplement: Supplementary Information [file srep35264-s1.doc]

CRISPR/Cas9 mediated knockout of *rb1* and *rbl1* leads to rapid and penetrant retinoblastoma development in *Xenopus tropicalis*

Naert Thomas^1^, Robin Colpaert^1^, Tom Van Nieuwenhuysen^1^, Dionysia Dimitrakopoulou^1^, Jannick Leoen^2^, Jurgen Haustraete^2^, Annekatrien Boel^3^, Wouter Steyaert^3^, , Trees Lepez^4^, Dieter Deforce^4^, Andy Willaert^3^, David Creytens^5^ , Kris Vleminckx^1,3^*

**^1^** Developmental Biology Unit, Department of Biomedical Molecular Biology, Ghent University, Ghent, Belgium

**^2^** Inflammation Research Center, VIB, Ghent, Belgium

**^3^** Center for Medical Genetics, Ghent University and Ghent University Hospital, Ghent, Belgium

^4^ Laboratory for Pharmaceutical Biotechnology, Ghent University, B-9000 Ghent, Belgium

^5^ Department of Pathology, Ghent University and Ghent University Hospital, Ghent, Belgium

**Supplemental Figures**


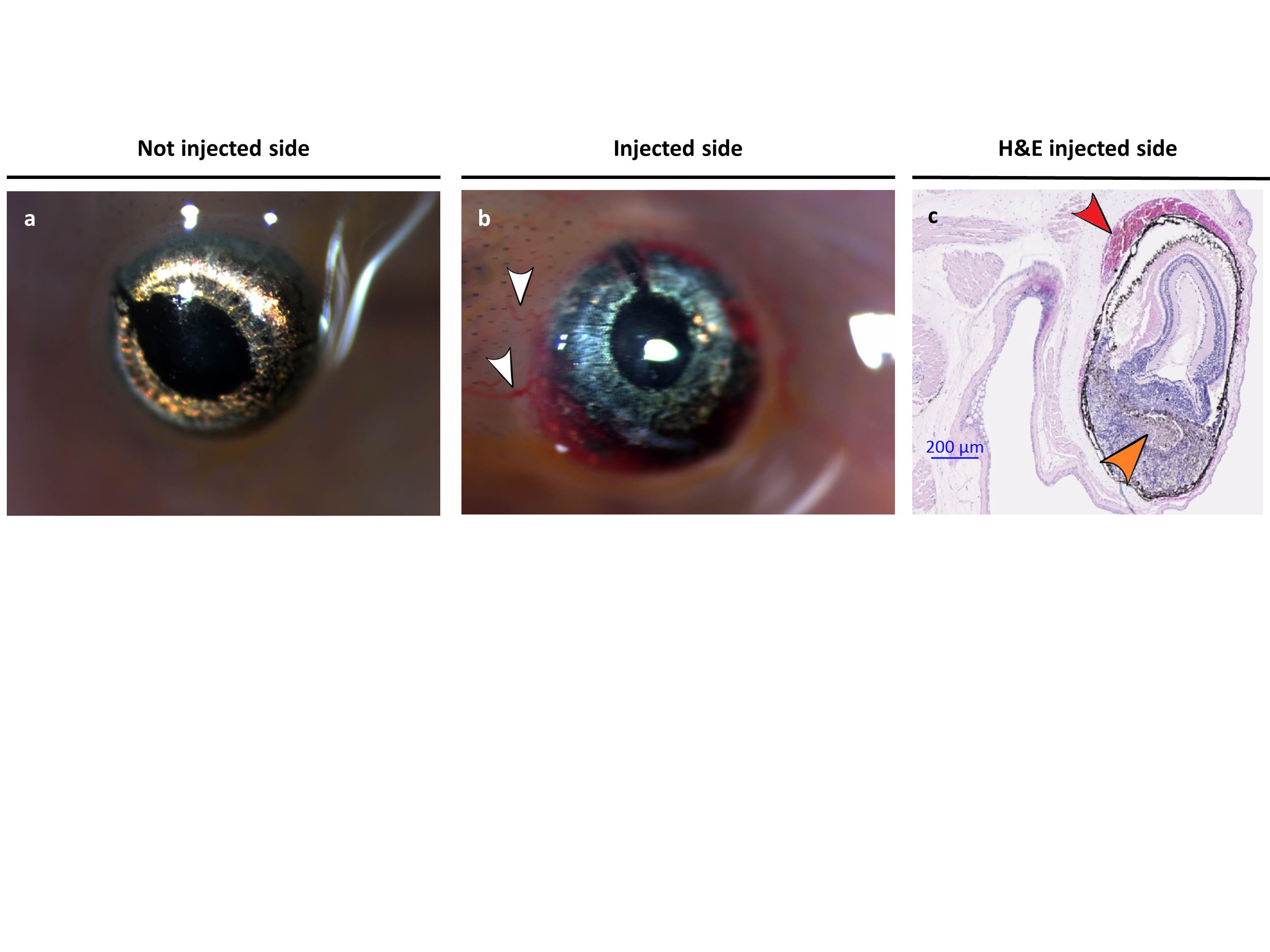


**Fig. S1:** **Retinoblastoma exhibiting tumor-associated neovasculature. (a)** The eye on the not injected side does not show any vasculature around the eye. **(b)** This whilst the eye on the injected side shows neovasculature feeding a retinoblastoma (white arrowheads). **(c)** H&E stained tissue section of injected eye shows neovasculature near the retinoblastoma (red arrowhead). Central area of necrosis within the retinoblastoma can also be observed (orange arrowhead).


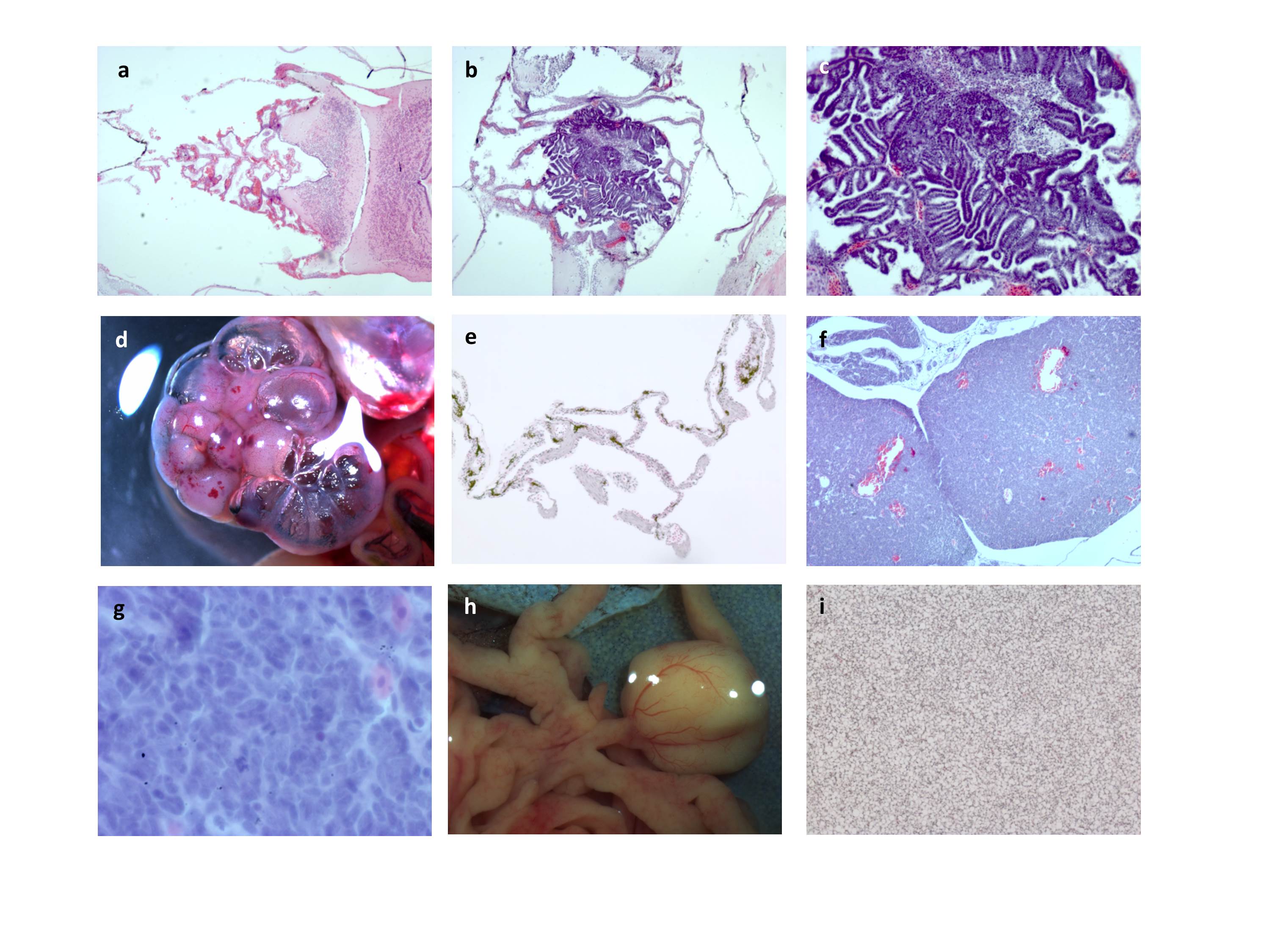


**Fig. S2: Array of neoplasms detected within rb1/rbl1 mosaic mutant animals**. **(a)** H&E stained control section of an choroid plexus from a wild-type animal. **(b-c)** H&E stained section of a choroid plexus neoplasm. **(d)** Lung tumor as observed during necropsy. **(e)** Control H&E stained section of the lung taken from the non-injected side of the animal as shown in Fig. S1d. **(f-g)** H&E stained section of the lung tumor as detected in Fig. S1d. **(h)** Hibernoma (white arrowhead) as observed during necropsy. **(i)** H&E stained section of the hibernoma.


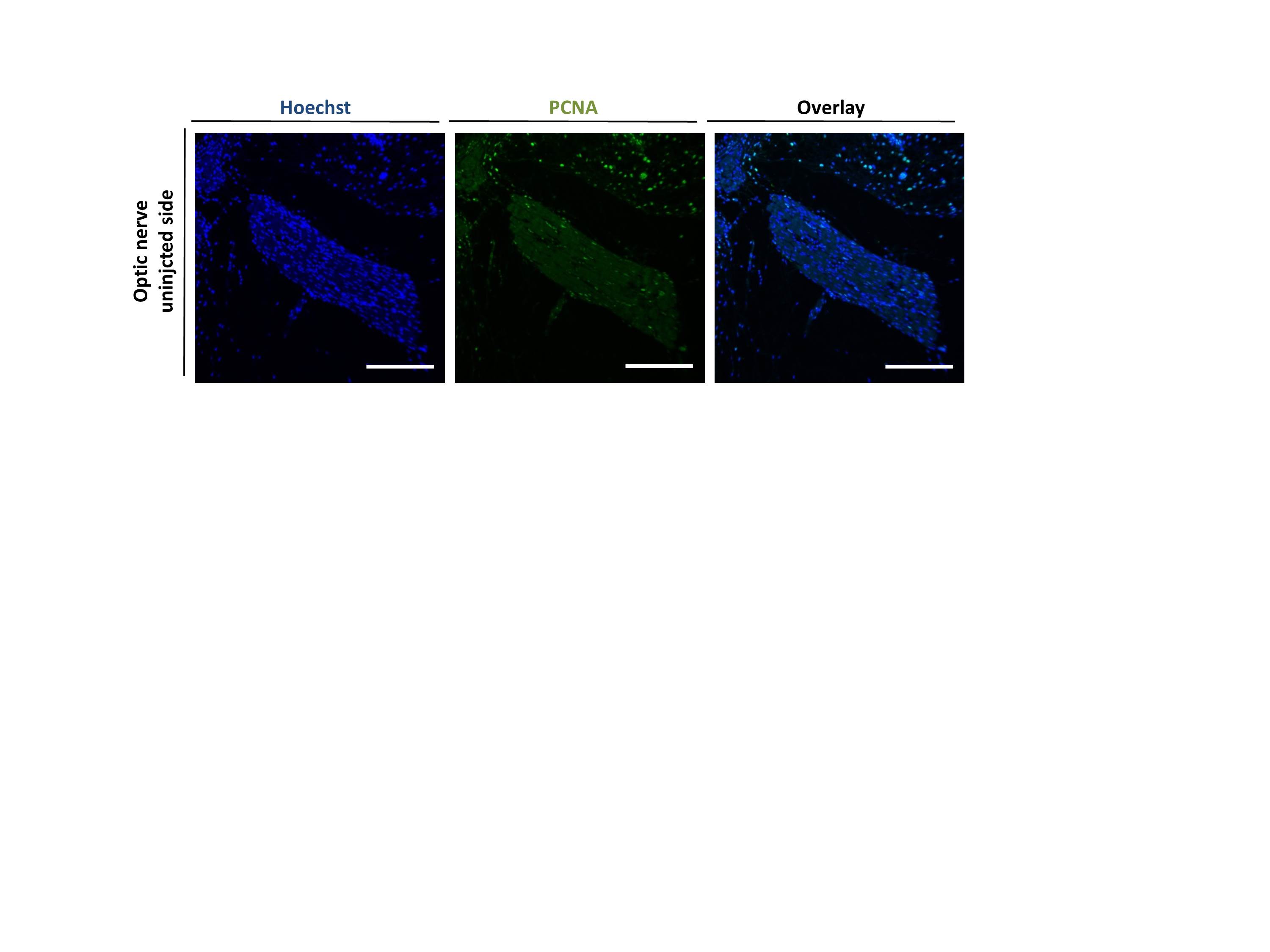


**Fig. S3: Proliferating cell nuclear antigen (PCNA) stain of an optic nerve of an eye on the not-injected side of a tadpole does not show masses of PCNA-positive cells within the nerve.** White scale bars corresponds with 200µM.


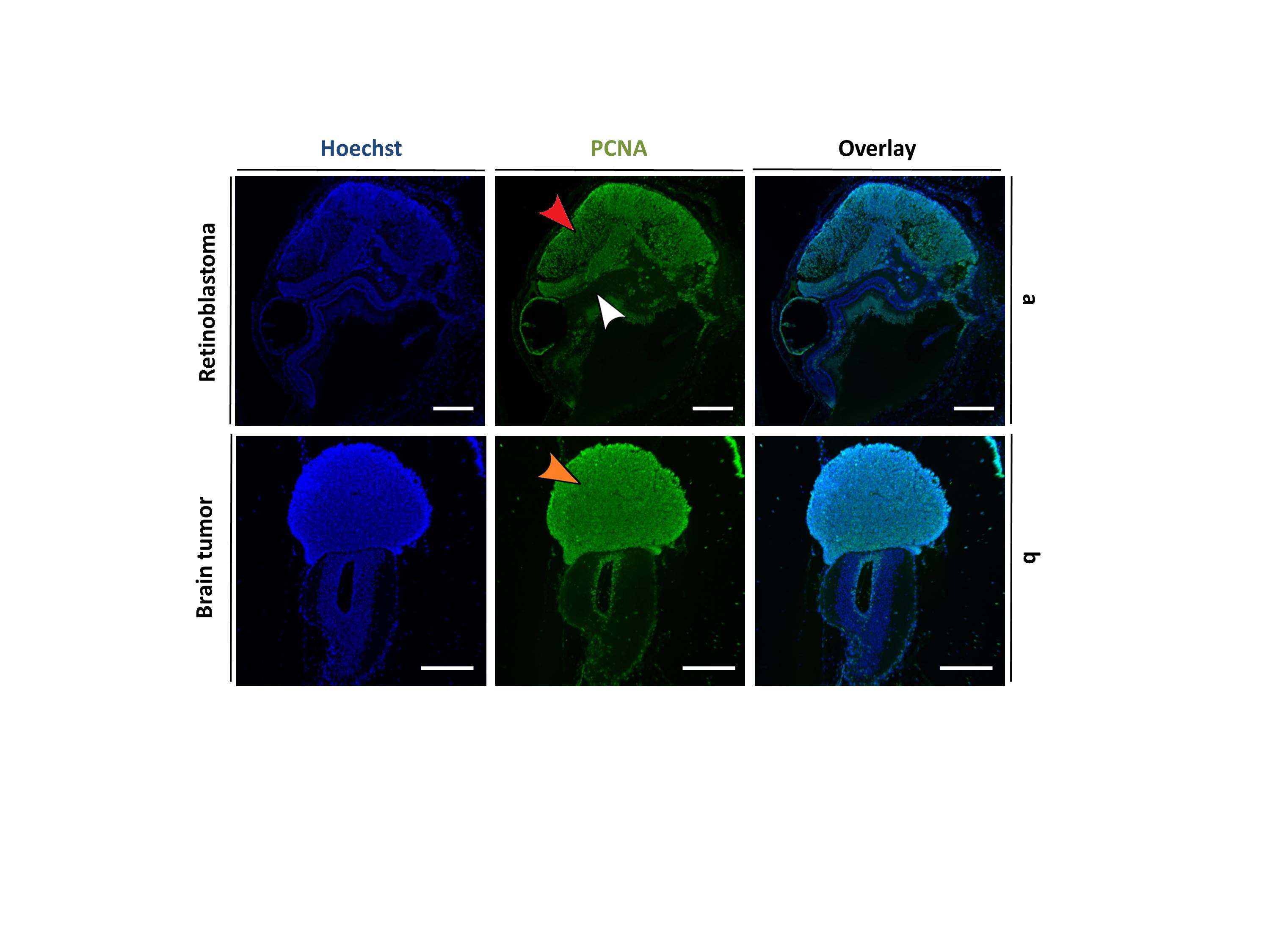


**Fig. S4: Proliferating cell nuclear antigen (PCNA) stain of the retinoblastoma and the brain tumor of an *rb1^cr2^/rbl1^cr2^* injected tadpole. (a)** Retinoblastoma shows clear PCNA immunostaining (red arrowhead) whilst the normal retinal layers remain quiescent (white arrowhead). **(b)** Brain tumor shows clear PCNA immunostaining (orange arrowhead) whilst the surrounding remaining brain tissue remains quiescent, with the exception of the subventricular proliferation zone. White scale bars correspond with 200µM.


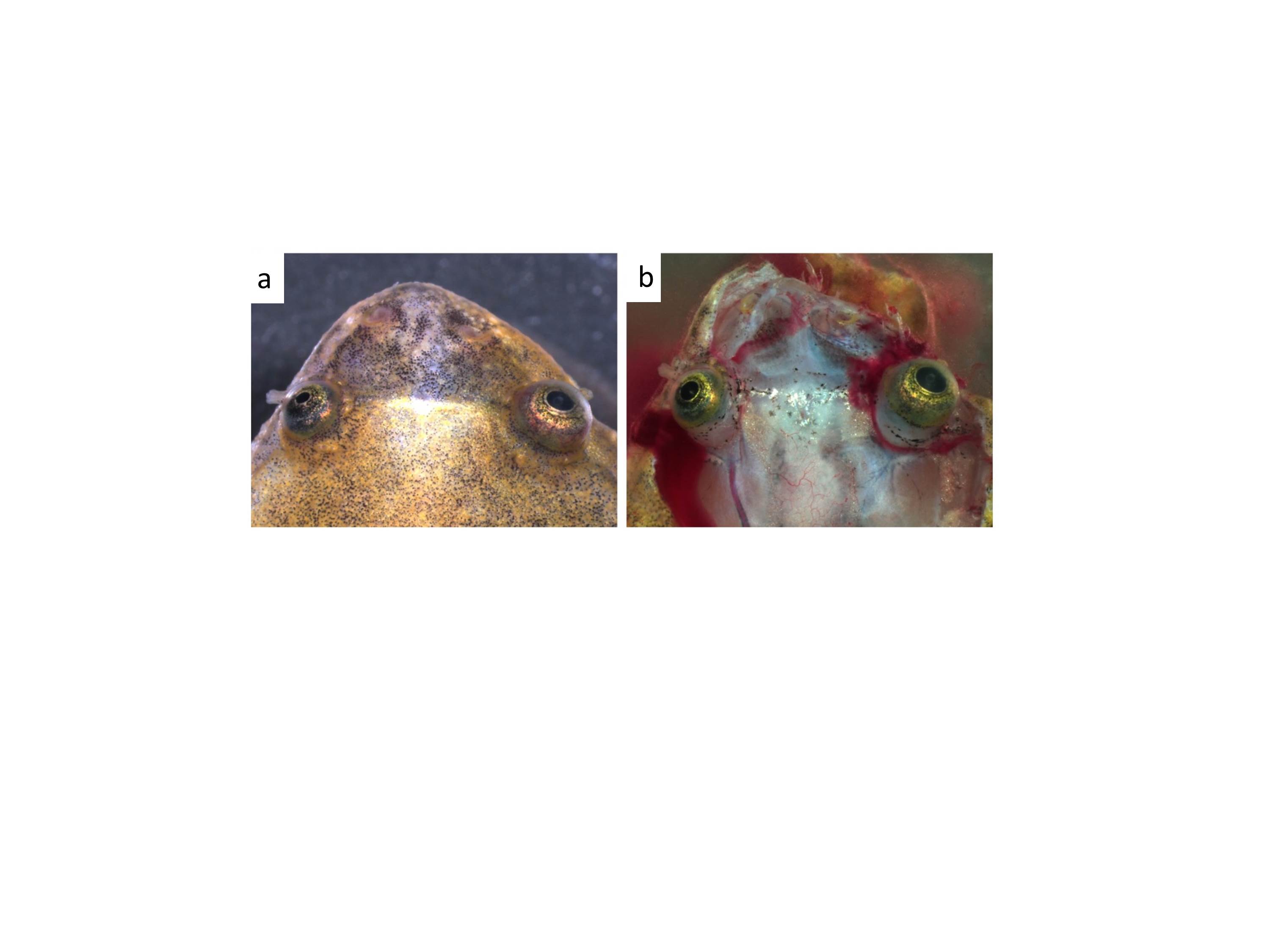


**Fig. S5: MDKO tadpole from which normal appearing eye and retinoblastoma was dissected for genetic analysis. (a)** The left eye appears normal whilst the right eye has developed a retinoblastoma. **(b)** Dissecting away the skin of this euthanized froglet reveals clear unilateral eye expansion.


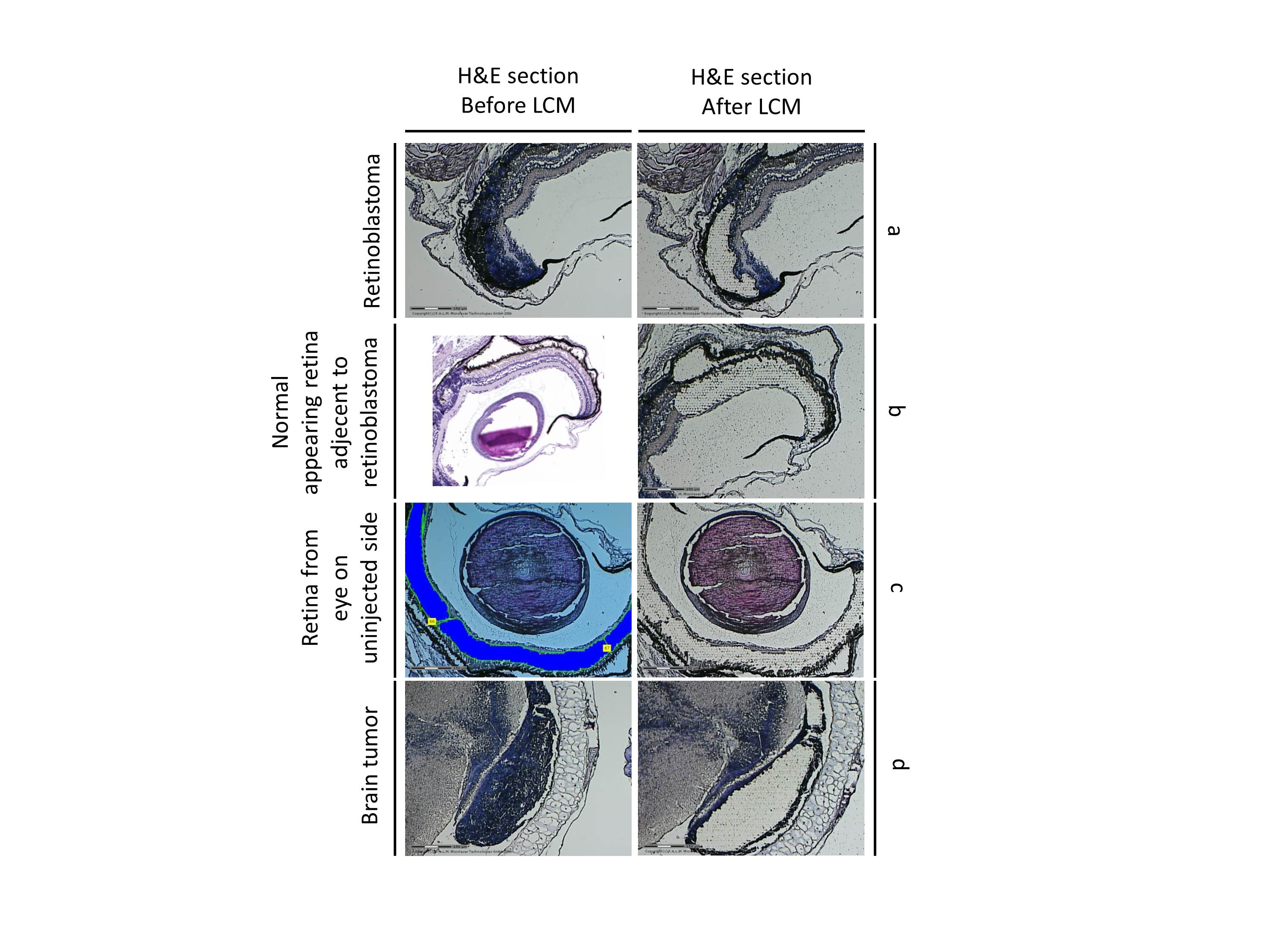


**Fig. S6: Laser capture micro dissection (LCM) method was employed to isolate specific cells from an H&E stained histological section taken from an *rb1^cr1^/rbl1^cr1^* injected tadpole.** All these cells are collected from slides from the same tadpole. **(a)** Isolation of retinoblastoma cells. **(b)** Isolation of normal appearing retina next to the retinoblastoma. **(c)** Isolation of retina from the eye on the not injected side. **(d)** Isolation of brain tumor.


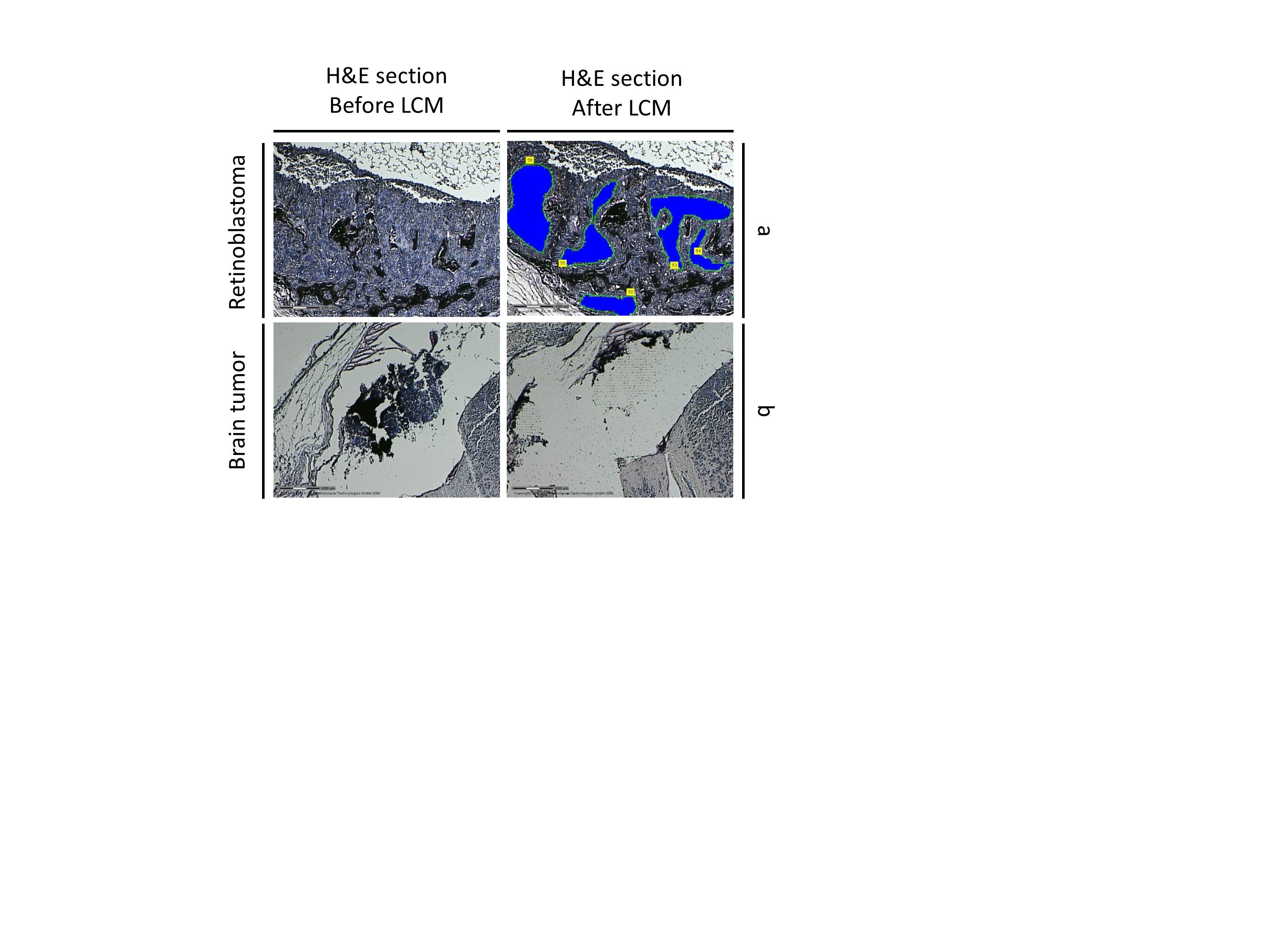


**Fig. S7: Laser capture micro dissection (LCM) method was employed to isolate specific cells from an H&E stained histological section taken from an *rb1^cr1^/rbl1^cr1^* injected tadpole.** All these cells are collected from slides from the same tadpole. **(a)** Isolation of retinoblastoma cells whilst attempting not to transfer any pre-existent cells. **(b)** Isolation of brain tumor.


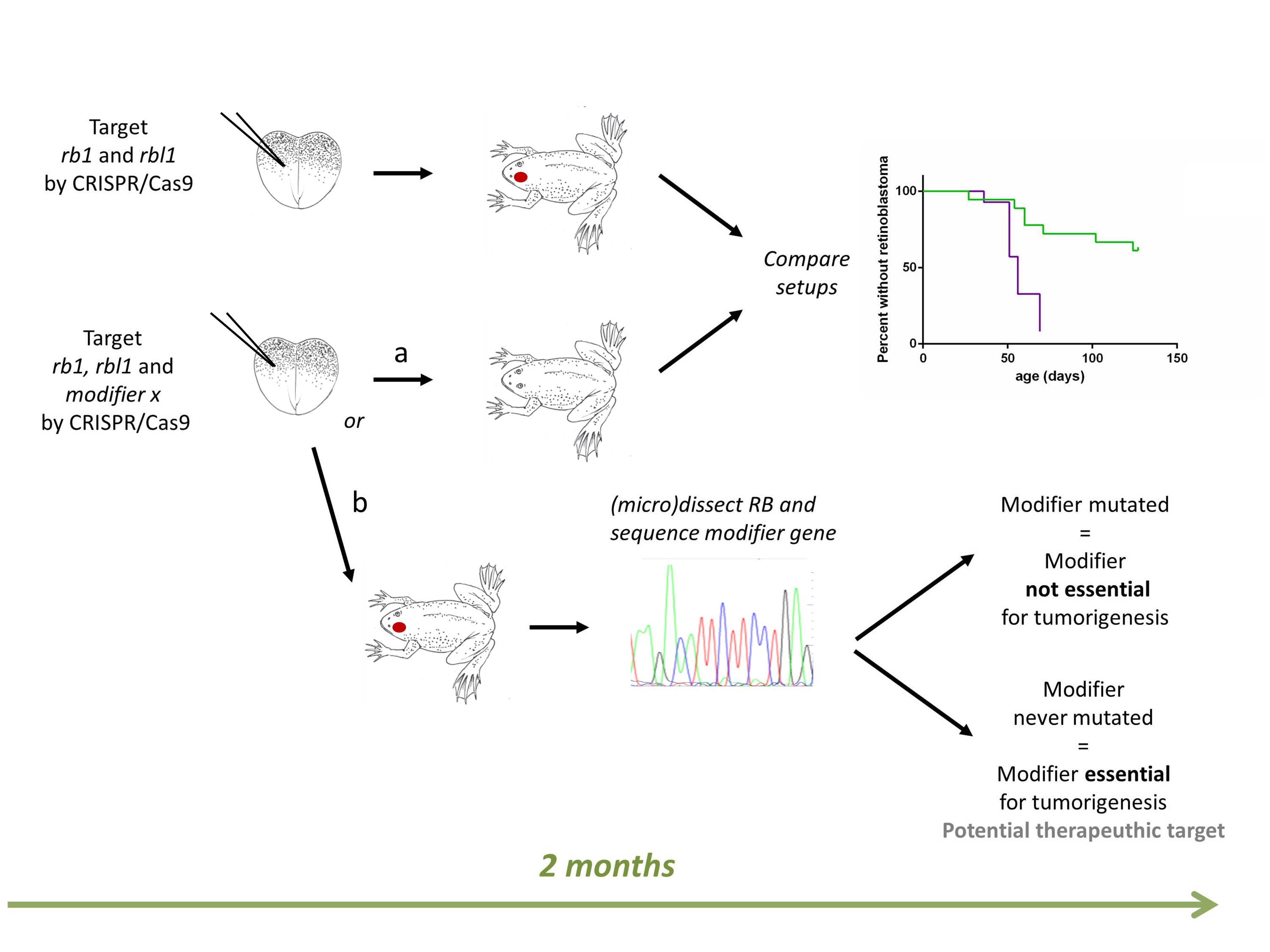


**Fig. S8: Rapid (2 months) and semi high-throughput identification of retinoblastoma therapeutic targets by triple multiplex CRISPR/Cas9. (a)** Targeting a modifier, in addition to *rb1* and *rbl1*, might influence the incidence of retinoblastoma or the survival of triple mosaic knockout animals when compared with *rb1/rbl1* double mosaic knockout animals. **(b)** Retinoblastoma developing in triple knockout animals can be (micro)dissected and the modifier gene locus sequenced. If the modifier gene is never mutated within the retinoblastoma, but efficient genome editing of this locus in control tissue has been shown, this provides evidence that the modifier is essential for tumorigenesis. This modifier then represents an attractive drug target for targeted therapy.

**Supplemental Tables**

**Table S1: Raw data output for all the targeted deep sequencing discussed within this paper – see accompanying Excell file.**

| **Setup** | gRNA1 (pg) | gRNA2 (pg) | gRNA3 (pg) | Cas9 (pg) |
| --- | --- | --- | --- | --- |
| *rb1^cr1^* | 1000 | n.a. | n.a. | 1000 |
| *rbl1^cr1^* | 1000 | n.a | n.a. | 1000 |
| *rb1^cr1^* + *rbl1^cr1^* | 445 | 350 | n.a. | 500 |
| *rb1^cr2^* + *rbl1^cr2^* | 650 | 800 | n.a. | 500 |
| *rb1^cr2^* + *rbl1^cr2^* + *syk1* | 200 | 200 | 200 | 500 |

**Table S2: Concentrations of sgRNAs and Cas9 as present in the 1 nl injection volume microinjected unilaterally in two-cell *Xenopus tropicalis* embryos.**

|  | **Forward primer** | **Reverse primer** |
| --- | --- | --- |
| ***rb1^cr1^*** | 5’-TTTGGAAGGACTAACGAC-3’ | 5’-TGGGAGTCAATTCTTAGG-3’ |
| ***rbl1^cr1^*** | 5’-GCTCTGTTCCTTCATTGTTCC-3’ | 5’-GGCTTATTAGTTGTTCCTTCTG-3’ |
| ***rb1^cr2^*** | 5’-AGACAAACAAGGGAACGGGA-3’ | 5’-TGAAGGCTGAAGGTTTGCTG-3’ |
| ***rbl1^cr2^*** | 5’-CCCTTAGCTCAACACATATGG-3’ | 5’-GAAACAGAGAACCTACATCGCC-3’ |
| ***syk*** | 5’-GGGCATTTGGATTACTTTCTG-3’ | 5’-TTTGAGCACAGGTTTGTAACAC-3’ |

**Table S3: Primer pairs used to amplify the CRISPR/Cas9-targeted sequences from genomic DNA.**

**Supplemental Materials and Methods**

*Recombinant NLS-Cas9-NLS generation and extraction.*

recombinant NLS-Cas9-NLS was expressed in the Escherichia coli strain BL21codon + pICA2 after transformation with pLHM36NLS(S)-Cas9(SP)-NLS(N), respectively, in which expression is induced by isopropyl β-D-1- thiogalactopyranoside (IPTG) under control of a pL-promoter developed by the Protein Service Facility of VIB (WO 98/48025). The pLHM36 plasmid is provided with a His_6_-Maltose binding protein (MBP) tag at the N-terminus followed by a murine caspase-3 site. The murine caspase-3 site can be used for the removal of the His_6_-MBP tag during purification. The NLS(S)-Cas9(SP)-NLS(N) protein is a fusion of the NLS sequence in the SV40 large T-antigen followed by the CRISPR-associated endonuclease Cas9/Csn1 protein from S. pyogenes and the NLS sequence from nucleoplasmin. The transformed bacteria were grown in Luria Bertani medium supplemented with ampicillin (100 μg/mL) and kanamycin (50 μg/mL) overnight at 28 °C before 1/100 inoculation in a 20-L fermenter provided with Luria Bertani medium supplemented with ampicillin (100 μg/mL) and 1% glycerol. The initial stirring and airflow was 200 rpm and 1.5 L/min., respectively. This was further automatically adapted to keep the pO_2_ at 30%. The temperature was kept at 28 °C. The cells were grown to an optical density of 1.0 as measured by the absorbance at 600 nm, hold at 28 °C, and expression was induced by addition of 1 mM IPTG overnight. Cells were then harvested and frozen at -20 °C. After thawing, the cells were resuspended at 3 mL/g in lysis buffer 50 mM Tris(hydroxymethyl)aminomethane (Tris) pH 8.0, 1 M NaCl, 1 mM 1,4-Dithiothreitol (DTT) and 1 tablet Complete EDTA-free(Roche)/50 mL. The cytoplasmic fraction was prepared by sonication of the cells and was isolated by centrifugation at 18,000 x g for 30 min. All steps were conducted at 4 °C. During sample preparation, DNA was removed by polyethyleneimine (PEI) precipitation where 0.25 % PEI was added, the supernatant was recovered by centrifugation. Further, the supernatant was also treated with 70 % ammonium sulphate during 20 minutes and the protein pellet was recovered by centrifugation. This protein pellet was resuspended in lysis buffer for further purification on Ni-Sepharose 6 FF column (GE Healthcare), equilibrated with 2 x phosphate buffer saline (PBS), 20 mM imidazole and 1 mM DTT. The column was eluted with 2 x PBS, 250 mM imidazole, 10 % glycerol and 1 mM DTT after an extra wash step with 6 x PBS. The elution fraction was diluted with 20 mM Hepes pH 7.5, 10 % glycerol, 1 mM DTT and 100 mM KCl to a concentration of 0.25 mg/ml before 1/100 m/m murine caspase-3 was added to remove the His_6_-MBP fusion during 1 hour and 30 minutes at 30°C. This solution (after dilution with 20 mM Hepes pH 7.5, 10 % glycerol to a conductivity of 10 mS/cm) was further purified on Capto S with 20 mM Hepes pH 7.5, 10 % glycerol, 1 mM DTT, 100 mM KCl as equilibration buffer and 20 mM Hepes pH 7.5, 10 % glycerol, 1 mM DTT, 1 M KCl as elution buffer. A gradient over 2.5 column volumes was used. This step was performed to remove murine caspase-3 and the His_6_-MBP fusion that stayed in the flow-through of the column. Finally, the purified protein was dialyzed to 20 mM Hepes pH 7.5, 150 mM KCl, 10 % glycerol; the purity was analyzed by SDS-PAGE and the concentration was determined using the Micro-BCA assay.
